# *Candidatus* Neoehrlichia mikurensis and its co-circulation with *Anaplasma phagocytophilum* in *Ixodes ricinus* ticks across ecologically different habitats of Central Europe

**DOI:** 10.1186/1756-3305-7-160

**Published:** 2014-04-02

**Authors:** Markéta Derdáková, Radovan Václav, Lucia Pangrácova-Blaňárová, Diana Selyemová, Juraj Koči, Gernot Walder, Eva Špitalská

**Affiliations:** 1 Institute of Zoology, Slovak Academy of Sciences, Dúbravská cesta 9, 845 06 Bratislava, Slovakia; 2 Institute of Parasitology, Slovak Academy of Sciences, Košice, Slovak Republic; 3 Institute of Virology, Slovak Academy of Sciences, Bratislava, Slovak Republic; 4 Section of Hygiene and Medical Microbiology, Innsbruck Medical University, Innsbruck, Austria

**Keywords:** *Candidatus* Neoehrlichia mikurensis, *Anaplasma phagocytophilum*, *Ixodes ricinus*, Human granulocytic anaplasmosis, Neoehrlichiosis

## Abstract

**Background:**

*Candidatus* Neoehrlichia mikurensis is a newly emerging tick-borne bacterium from the family Anaplasmataceae. Its presence in *Ixodes ricinus* ticks was reported from various European countries, however, it’s ecology and co-circulation with another member of the same family, *Anaplasma phagocytophilum* has not been rigorously studied yet.

**Findings:**

*Candidatus* N. mikurensis was detected in all sampling sites. In total, 4.5% of ticks were positive including larvae. The highest positivity was detected in Austria with a prevalence of 23.5%. The probability of *Candidatus* N. mikurensis occurrence increased with the proportion of ticks infected with *Anaplasma phagocytophilum*.

**Conclusion:**

A positive association between the occurrences of *Candidatus* N. mikurensis and *A. phagocytophilum* indicates that both bacteria share similar ecology for their natural foci in Central Europe.

## Findings

In Europe, *Candidatus* N. mikurensis represents a newly emerging tick-borne zoonotic bacterium from the family Anaplasmataceae. Phylogenetic analyses revealed that it is closely related to the *Ehrlichia*-like microorganisms previously detected in ticks and rodents from various regions of Europe and Asia [1-6]. Recently its pathogenicity was reported, as it was detected in immunosuppressed patients with septicaemia [7-9]. Rodents are the competent reservoir hosts since they develop a systemic infection [1,3,4,6] and are able transmit *Candidatus* N. mikurensis to the xenodiagnostic ticks [10]. The prevalence of *Candidatus* N. mikurensis in ticks over Europe varies, usually not exceeding 10%. Most reports are from Western Europe [2,4,5,11]. Recently it was reported in questing *I. ricinus* from Hungary [12] and Austria [13]. Here we report the prevalence of *Candidatus* N. mikurensis from 11 diverse ecological habitats from three Central European countries and its co-circulation in natural foci with *Anaplasma phagocytophilum*.

**Figure 1:**
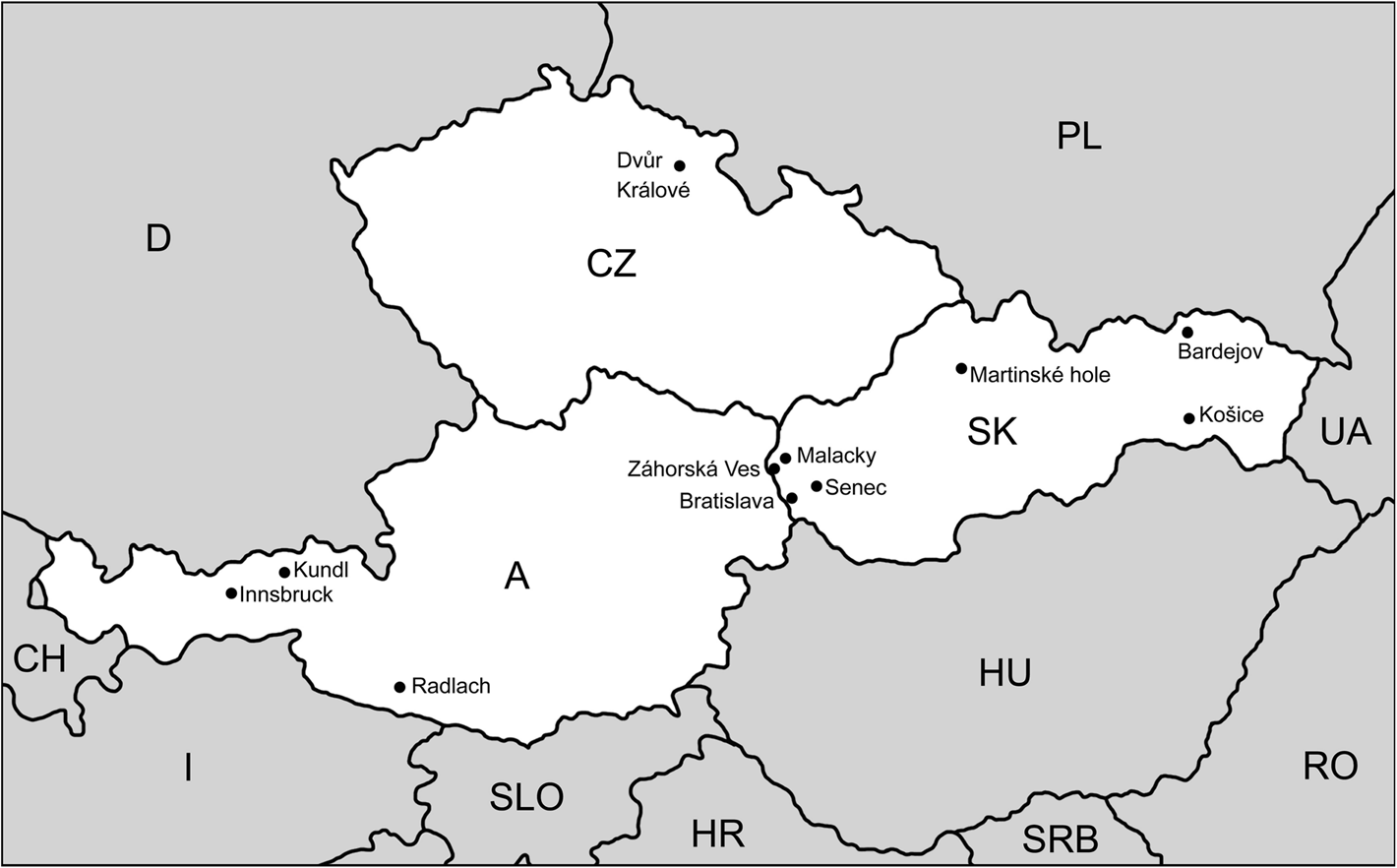
Map of sampling sites from Austria (A), Czech Republic (CZ) and Slovakia (SK); Central Europe.

A total of 1535 (755 adults, 614 nymphs, 140 larvae, and 26 individuals for which the developmental stage was not identified) and 1413 (756 adults, 621 nymphs, 10 larvae, and 26 individuals for which the developmental stage was not identified) *I. ricinus* ticks from three Central European countries (Slovakia, the Czech Republic and Austria) (Figure 1) were tested for the presence of *Candidatus* N. mikurensis and *A. phagocytophilum*, respectively. Ticks were sampled from diverse habitats (Table 1) by blanket dragging. DNA was extracted from single individuals by DNeasy Blood & Tissue Kit (Qiagen, Hilden, Germany). The presence of *Candidatus* N. mikurensis was detected by RT-PCR of *groEL* gene as described before [4] or by nested PCR of the specific fragment of 16S *rRNA* gene [1]. *A. phagocytophilum* was detected by RT-PCR of *msp2* according to a previously described protocol [14] or by nested PCR amplifying the specific 546 bp fragment of 16S *rRNA*[15].

**Table 1:** **Prevalence of *Candidatus *** **N. mikurensis (CNM) and *A. phagocytophilum *** **(AP) in *I. ricinus *** **ticks from sampling sites in Slovakia, the Czech Republic and Austria**

	**Geographical coordinates**	Number of ticks tested*	***Ca. *** **N. mikurensis positive (%)**	***A. phagocytophilum *** **positive (%)**	**Habitat type and altitude**
Bratislava (SK)	48°10′N 17°04′E	378/248	4 (1.1)	10 (4)	Oak-beech, suburban and urban forests
Senec (SK)	48°16′N 17°21′E	97	6 (6.2)	1 (1)	Native fragmented, dry oak forest
Malacky (SK)	48°26′N 17°01′E	93/101	2 (2.2)	4 (4)	Urban park with maples, oak hornbeam
Záhorská Ves (SK)	48°22′N 16°53′E	121	14 (11.6)	5 (4.1)	Farmland and pine lowland forest
Martinské hole (SK)	49°05′N 18°51′E	219	7 (3.2)	6 (2.7)	Mountain spruce forest
Košice (SK)	48°44′N 21°16′E	224	6 (2.7)	10 (4.5)	Oak-hornbeam urban forest
Bardejov (SK)	49°19′N 21°16′E	179	8 (4.5)	3 (1.7)	Oak, beech, maple, birch suburban
Dvur Kralove (CZ)	50°25′N 15°48′E	138	3 (2.2)	8 (5.8)	Mixed and pine suburban forest
Austria total		86	19 (22.1)	6 (7.0)	
Innsbruck (AT)	47°17′N 11°26′E	26	5 (19.2)	3 (11.5)	Mountain fir forest
Kundl (AT)	47°28′N 11°60′E	51	12 (23.5)	3 (5.9)	Beech-fir forest
Radlach (AT)	46°45′N 13°15′E	9	2 (22.2)	0 (0)	Alder and ash forest
Total		1535/1413	69 (4.5)	53 (3.8)	

*If different numbers of ticks were analyzed for the presence of CNM and AP, two values (CNM/AP) are shown for the site (for Bratislava, 130 larvae were included in analysis for the presence of NM, but they were excluded in analysis for the presence of AP; for Malacky, not all ticks tested for CNM were tested for the presence of AP due to the lack of DNA.

*Candidatus* N. mikurensis was detected in all 11 sampling sites. In total, 69 (4.5%) of 1535 ticks were positive. The prevalence of *Candidatus* N. mikurensis ranged from 1.1% to 23.5% (Table 1). In Austria at the site Kundl, *Candidatus* N. mikurensis was detected in 4 of 10 questing larvae.

In Total, 1413 ticks were tested for *A. phagocytophilum* and 53 (3.8%) were positive. It was detected in all sampling sites, but one (Table 1). None of the tested larvae carried *A. phagocytophilum*.

Furthermore, we analyzed the relationship between the occurrence probability of *Candidatus* N. mikurensis and the proportion of ticks infected with *A. phagocytophilum* with a generalized linear mixed model (GLMM). The number of ticks infected with *Candidatus* N. mikurensis was entered as a dependent variable and was linked with a binomial error to the number of all ticks from a given site and tick developmental stage. The proportion of ticks infected with *A. phagocytophilum* and the developmental stage of ticks were examined as fixed factors. As ticks for each site were examined at two developmental stages (nymphs and adults), site identity was entered as a random factor; three sampling sites from Austria were pooled due to sample size limitation. The probability of tick infection with *Candidatus* N. mikurensis increased with the proportion of ticks infected with *A. phagocytophilum*. The occurrence probability of *Candidatus* N. mikurensis did not differ between adult and nymphal ticks (Table 2). The solutions of random effects revealed that the occurrence probability of *Candidatus* N. mikurensis for the Austrian sites was significantly higher than the mean occurrence probability (estimate ± SE = 1.08 ± 0.43, t_6_ = 2.53, p = 0.039).

**Table 2:** GLMM analysis on the occurrence probability of CNM in questing ticks as a function of the proportion of ticks infected with AP and tick developmental stage

**Parameter**	**Estimate**	**SE**	**df**	**t**	**p**
Random effect					
Site ID	0.49	0.32			0.128
Fixed effects					
Intercept	-3.33	0.37	8	-8.96	< 0.001
Proportion of ticks infected with AP	7.02	2.69	7	2.61	0.035
Tick developmental stage_adults	-0.01	0.30	7	-0.01	0.996
Tick developmental stage_nymphs	0				

The pseudo-likelihood function was used to calculate parameter estimates. The analysis was conducted with SAS (SAS Institute Inc., Cary, NC) and the GLIMMIX macro.

We have confirmed the permanent circulation of *Candidatus* N. mikurensis and *A. phagocytophilum* in each of the three examined countries of Central Europe across a wide ecological spectrum of habitats (Table 1). The highest prevalence of *Candidatus* N. mikurensis (23.5%) was observed in Austria. A similarly high prevalence (24.2-26.6%) was found for questing ticks from Germany [16]. These are so far the highest prevalence results reported for Europe. Moreover, in Austria we have detected four positive questing larvae. Up to this date, the transovarial transmission has not been reported for *Candidatus* N. mikurensis. However, to our knowledge the questing larvae were examined for the pathogen only at one site in The Netherlands, by Jahfari *et al*. [4]. As the mode of pathogen transmission by vectors is of high epidemiological significance [17], possible transovarial transmission of *Candidatus* N. mikurensis should be elucidated in future studies.

The reservoir competency of rodents for *Candidatus* N. mikurensis have been recently confirmed [10]. As for *A. phagocytophilum*, it is unlikely that rodents are important reservoir hosts of the genotypes that are transmitted by *I. ricinus*. Based on the phylogenetic analyses of several genes, rodents in Europe are infected with distinct genotypes from that found in questing *I. ricinus* ([18], unpublished observation). Moreover, recent study showed that rodents infected with *A. phagocytophilum* were not able to transmit it to xenodiagnostic larvae [10]. The reservoir competence of other hosts for *Candidatus* N. mikurensis needs to be elucidated, since it was detected in the ticks feeding on red deer, mouflon and wild boar [4].

## Conclusions

We have revealed a positive association between the occurrences of *Candidatus* N. mikurensis and *A. phagocytophilum*. This finding indicates that both bacteria share similar ecology for their natural foci in Central Europe. This result has an important implication for public health, and patients with a history of tick bite should also be examined for the presence of *Candidatus* N. mikurensis since it is widespread throughout Central Europe in all regions where *I. ricinus* is present.

## Competing interests

The authors declare that they have no competing interests.

## Authors’ contributions

MD designed the study, collected the ticks, extracted DNA of ticks, analysed the presence of pathogens and has written the manuscript, RV has performed statistical analyses and helped with the writing of the manuscript, LPB, DS, JK, GW and ES collected ticks, extracted DNA of ticks and did the molecular analyses of ticks. All authors have read and agreed with the content of the final version of the manuscript.
